# Androgen-induced gut dysbiosis disrupts glucolipid metabolism and endocrinal functions in polycystic ovary syndrome

**DOI:** 10.1186/s40168-021-01046-5

**Published:** 2021-05-06

**Authors:** Qixin Han, Juan Wang, Weiping Li, Zi-Jiang Chen, Yanzhi Du

**Affiliations:** 1 Center for Reproductive Medicine, Ren Ji Hospital, School of Medicine, Shanghai Jiao Tong University, Shanghai, 200135 China; 2 Shanghai Key Laboratory for Assisted Reproduction and Reproductive Genetics, Shanghai, 200135 China; 3 Center for Reproductive Medicine, Cheeloo College of Medicine, Shandong University, Jinan, 250012 Shandong China; 4 National Research Center for Assisted Reproductive Technology and Reproductive Genetics, Shandong University, Jinan, 250012 Shandong China; 5 Key laboratory of Reproductive Endocrinology of Ministry of Education, Shandong University, Jinan, 250012 Shandong China

**Keywords:** Polycystic ovary syndrome, Gut microbiota, 16S rRNA gene sequence, Metabolome, Faecal microbiota transplantation

## Abstract

**Background:**

The characteristics of polycystic ovary syndrome (PCOS), a common reproductive endocrinal disorder, are high incidence, complicated aetiology and poor therapeutic effects. PCOS patients frequently exhibit gut dysbiosis; however, its roles in the regulation of metabolic and endocrinal balances in PCOS pathophysiology are not clear.

**Results:**

In this study, gut dysbiosis was reproduced in dehydroepiandrosterone (DHEA)-induced PCOS-like rats. An antibiotic cocktail was used to eliminate gut microbiota during DHEA treatment; however, depletion of the gut microbiota did not prevent the occurrence of PCOS phenotypes in DHEA-treated rats. DHEA-shaped gut microbiota transplanted to pseudo germ-free recipients trigged disturbances in hepatic glucolipid metabolism and reproductive hormone imbalance. The clinical features of PCOS may be correlated with the relative abundance of gut microbes and the levels of faecal metabolites in faecal microbiota transplantation (FMT) recipient rats.

**Conclusion:**

These findings indicate that androgen-induced gut microbiota dysbiosis may aggravate metabolic and endocrinal malfunction in PCOS.

Video Abstract

**Supplementary Information:**

The online version contains supplementary material available at 10.1186/s40168-021-01046-5.

## Background

Polycystic ovary syndrome (PCOS) is a prevalent reproductive endocrine disorder in women. It is characterised by irregular menses, infertility and hirsutism due to chronic oligo-anovulation and an excess of androgen. Owing to its relatively high prevalence (~ 6−20%), PCOS places heavy social and economic burdens on patients [1, 2]. Subsequent morbidities, such as obesity, type 2 diabetes mellitus (T2DM), cardiovascular disease (CVD), endometrial carcinoma, pregnancy complications and depression significantly threaten patients’ physical and mental health and exert a far-reaching influence on their quality of life.

Currently, the 2003 Rotterdam Consensus is one of the most widely accepted diagnostic criteria for PCOS. It requires the presence of 2 or more of chronic anovulation, biochemical or clinical hyperandrogenism, or polycystic ovary morphology to establish PCOS. Coviello et al. reported that PCOS adolescents had a higher risk of metabolic syndromes related to hyperandrogenism regardless of insulin resistance and obesity [3]. Therefore, hyperandrogenism is considered a critical factor in both impaired follicular development and metabolic disorders in PCOS. However, it is still not completely understood how hyperandrogenism leads to increased PCOS metabolic syndrome susceptibility.

In the last 2 decades, the gut microbiota has emerged as an internal environmental factor closely related to human health. The colonisation of the gastrointestinal tract by enteric microorganisms leads to the participation of microbes in various physiological processes in the human body. Many studies have revealed that gender plays an important role in the composition of gut microbiota [4, 5]. The alpha diversity of gut microbiota is higher in females than in males [6, 7], with the divergence beginning at the onset of puberty in both humans and rodents, indicating that sex hormones induce compositional changes in the gut microbiome [8, 9]. Additionally, the gut microbiome is associated with the host metabolism. The microbial communities in individuals with obesity, T2DM and CVD were distinct from healthy counterparts [10–12]. Studies showed that faecal microbiota transplantation (FMT) led to the obese phenotype in germ-free mice. This finding added evidence to the causative role of the microbial community in the development of metabolic disorders [13].

The gut microbiome in PCOS patients shows an altered diversity, distinct species relative abundances and shifted functional enrichments [14–16]. Our previous study identified species-level differentially abundant microbes and found correlations between these metagenomic species and clinical parameters such as body mass index, serum testosterone (T), luteinising hormone (LH) and anti-Müllerian hormone levels [17]. Alterations in gut microbiota were often associated with hyperandrogenism and metabolic markers [16, 18]. The administration of pro- or prebiotics improved both reproductive and metabolic phenotypes, suggesting that the gut microbiome might be involved in PCOS pathogenesis [19, 20]. Recently, it was reported that gut microbiota transplantation from PCOS patients to animals could result in glucose intolerance, insulin resistance and a reduced number of pups in the first litter. It suggests that the gut microbiota plays a causal role in PCOS development. However, gut dysbiosis may be the result of numerous factors, such as hyperandrogenism, metabolic disorders or various lifestyle choices. Due to the genetic and environmental homogeneity of rats, the androgen-treated rat model helps to reveal the role of gut microbiota in the development of androgen-induced PCOS phenotypes. In this study, the dysbiosis of gut microbiota was validated in androgen-induced PCOS-like rats. Then, a PCOS-like animal model was established in pseudo germ-free rats. Finally, an androgen-shaped gut microbiome was transplanted to the pseudo germ-free rats to investigate the effects of the disrupted gut microbial community on the host. The potential mechanism underlying these processes was also discussed.

## Results

### DHEA-induced metabolic, ovarian and endocrinal disorders in Sprague Dawley (SD) rats

**Fig. 1:**
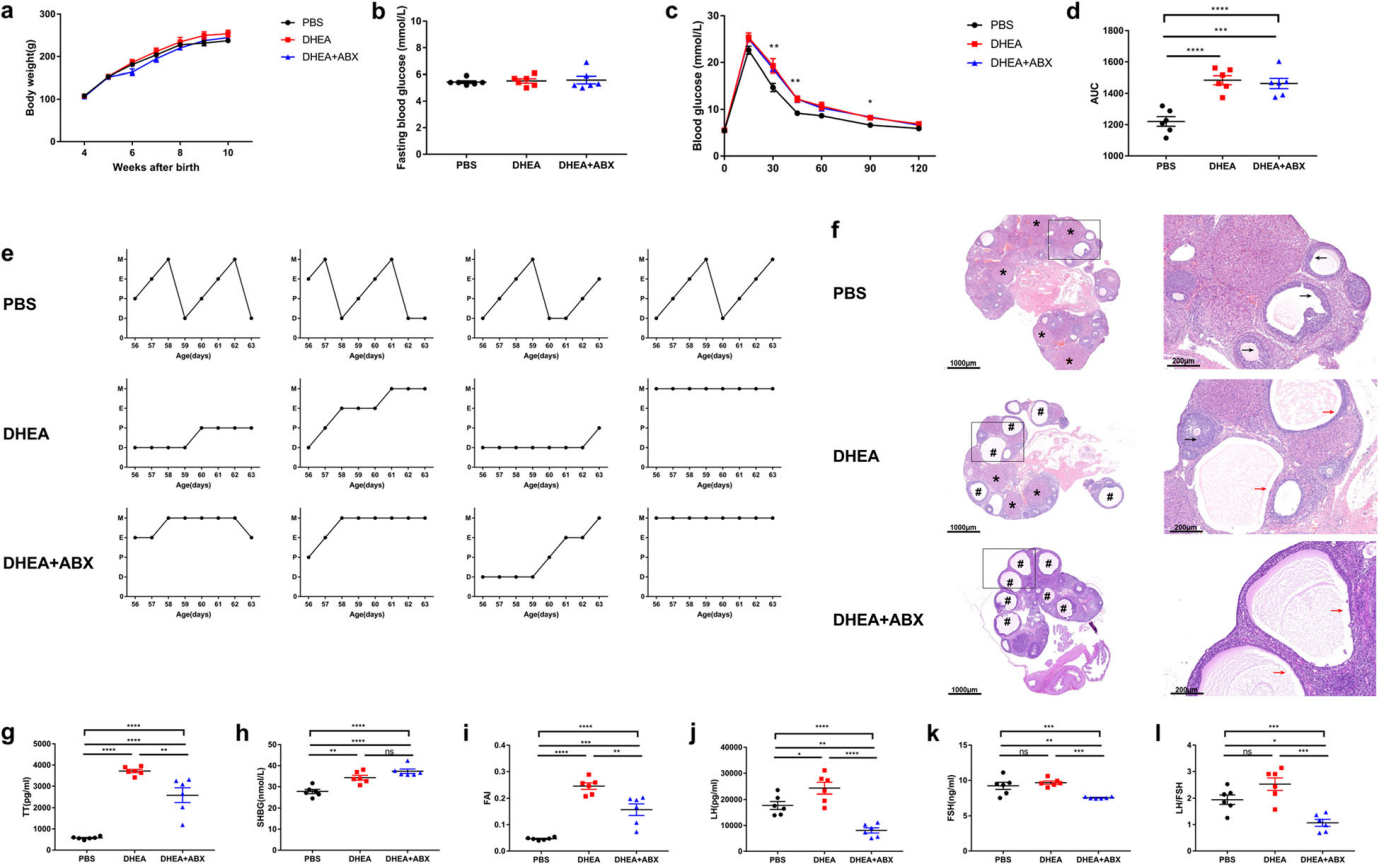
DHEA-induced glucose metabolism, ovarian morphology, and reproductive hormone level alterations in conventional and antibiotic-treated rats. **a** Bodyweight, **b** FBG, **c** GTT, **d** area under the curve of GTT, **e** oestrous cycle examination and **f** H&E staining of representative ovaries. * indicates CL, and cyst-like follicles are indicated by #. Black arrows indicate normal granulosa cell layers. Red arrows indicate attenuated granulosa cell layers. Scale bar = 1000 μm (left) and 200 μm (right). **g** serum TT levels, **h** serum SHBG levels, **i** FAI, **j** serum LH levels, **k** serum FSH levels and **l** LH:FSH ratio, DHEA + ABX, DHEA + antibiotics. Data are given as mean ± SEM. *n* = 6 rats per group. * *p* < 0.05; ** *p* < 0.01; *** *p* < 0.001; **** *p* < 0.0001

To induce a PCOS-like model, SD rats were injected subcutaneously with DHEA (6 mg/100 g bodyweight) daily for 35 days in the DHEA group, while control rats were injected with phosphate buffer saline (PBS). During the 5-week treatment, the weight of the DHEA-treated rats was similar to their PBS-treated counterparts (Fig. 1a) and the levels of fasting blood glucose (FBG) were similar between groups (Fig. 1b). Compared with PBS-treated rats, the DHEA-treated rats displayed higher glucose intolerance (Fig. 1c, d) and disrupted oestrous cycles (Fig. 1e). Ovaries from the PBS group contained follicles at different stages of development and several corpora lutea (CL). In contrast, those from the DHEA group showed multiple cyst-like follicles with collapsed walls and fewer CL **(**Fig. 1f). In addition, the levels of total testosterone (TT), sex hormone-binding globulin (SHBG), free androgen index (FAI), and LH were elevated in the DHEA-treated rats (Fig. 1g–j). The increased TT and FAI verified the efficiency of DHEA administration and the successful development of hyperandrogenic rats. However, no differences were observed in follicle-stimulating hormone (FSH) levels or the LH/FSH ratio (Fig. 1k, l). These results validate the successful establishment of a PCOS-like rat model with disorders in metabolism, ovarian morphology, and endocrine function.

### Hyperandrogenism in female SD rats was accompanied by gut microbiota dysbiosis

Faecal samples of PBS- and DHEA-treated rats were sequenced using the Illumina-MiSeq platform. Regarding alpha diversity, the phylogenetic diversity (PD) whole tree index significantly decreased in the gut microbiota of DHEA-treated rats compared with those of PBS-treated ones (Additional file 1: Figure S1a). Principal coordinate analysis (PCoA) based on the unweighted UniFrac distance did not show significant differences in clustering in faecal samples between the DHEA and PBS groups (Additional file 1: Figure S1b). At the genus level, the DHEA group was characterised by the decreased relative abundance of *Turicibacter*, *Anaerofustis* and *Clostridium sensu stricto* (Additional file 1: Figure S1c). From linear discriminant analysis effect size (LEfSe) analysis, the genus *Turicibacter*, family *Erysipelotrichaceae*, order *Erysipelotrichales,* class *Erysipelotrichia* was top-ranking by linear discriminant analysis (LDA) score in contributing to sample separation between groups (Additional file 1: Figure S1d, e).

### DHEA-induced PCOS-like phenotype in pseudo germ-free rats

Rats in both the DHEA group and DHEA + antibiotics group were injected subcutaneously with 6 mg/100 g bodyweight DHEA daily for 35 days. During this period, to establish pseudo germ-free rats, rats in the DHEA + antibiotics group received a freshly prepared antibiotic cocktail (ABX) containing 1 mg mL^−1^ ampicillin sodium, 1 mg mL^−1^ neomycin sulfate, 1 mg mL^−1^ metronidazole, and 0.5 mg mL^−1^ vancomycin hydrochloride in drinking water, while rats in the DHEA group were provided with non-acidified drinking water during the establishment of the PCOS model. 16S rRNA gene sequencing was performed to confirm the effects of ABX administration. There was a substantial decrease in alpha diversity in the DHEA + antibiotics group (Additional file 2: Figure S2a, b), indicating that ABX significantly suppressed the gut microbiota. The variation, confirmed by PCoA and analysis of similarities (ANOSIM) analyses, was higher between groups than within groups (Additional file 2: Figure S2c, d). These findings demonstrated that the ABX treatment provided a suitable environment for FMT. The body weight, FBG and glucose tolerance were not affected by the clearance of gut microbiota (Fig. 1a–d). Regarding the oestrous cycle, both groups were acyclic (Fig. 1e). Ovarian morphological anomalies, such as cyst-like follicles and thinner granulosa cell layers, were also observed in the DHEA + antibiotics group (Fig. 1f). These data suggest that the lack of a gut microbiome does not affect disorders in glucose metabolism and ovarian function in hyperandrogenic rats. Serum TT, FAI, LH, FSH, and the LH/FSH ratio decreased in the DHEA + antibiotics group compared with the DHEA group (Fig. 1g, i–l). However, serum SHBG remained unchanged between groups (Fig. 1h). The serum levels of reproductive hormones were significantly affected by gut microbiota clearance. In general, the presence of a gut microbial community may not be necessary for the development of the PCOS phenotype in DHEA-treated rats.

### Transplantation of DHEA-shaped gut microbiome leads to glucose intolerance, ovarian malfunction and endocrinal disturbance in pseudo germ-free rats

**Fig. 2:**
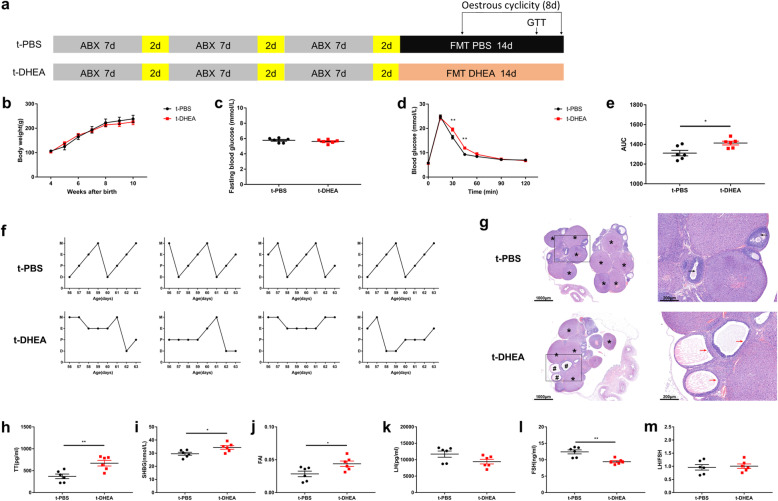
DHEA-shaped gut microbiota reproduced glucose intolerance, polycystic ovaries and reproductive hormone disorders in FMT recipients. **a** Timeline for animal experiments in FMT recipients. Yellow bars represent normal feeding with standard chow and non-acidified drinking water. Other treatments are indicated in the schema. **b** Bodyweight, **c** FBG, **d** GTT, **e** area under the curve of GTT, **f** typical oestrous cycles and **g** H&E staining of typical ovaries. * indicates CL, and cyst-like follicles are indicated by #. Black arrows indicate normal granulosa cell layers. Red arrows indicate attenuated granulosa cell layers. Scale bar = 1000 μm (left) and 200 μm (right). **h**  serum TT levels, **i** serum SHBG levels, **j** FAI, **k** serum LH levels, **l** serum FSH levels and **m** LH:FSH ratio. Data are given as mean ± SEM. *n* = 6 rats per group. * *p* < 0.05; ** *p* < 0.01

The timeline for the animal experiments with FMT recipients is shown in Fig. 2a. A three-course antibiotic cocktail was used for 27 days to reduce gut microbiota and to establish a pseudo germ-free rat model [21]. After ABX treatment, the gut microbial loads were significantly decreased (Additional file 3: Figure S3a), providing a favourable environment for the FMT. Subsequently, the rats were divided into 2 groups, an FMT recipient from the PBS group (t-PBS, *n* = 6), and an FMT recipient from the DHEA group (t-DHEA, *n* = 6). For the following 14 consecutive days, the pseudo germ-free rats in the t-PBS and the t-DHEA groups received a daily gavage of gut microbiota (0.1 g fecal pellet in 1 ml mixture of PBS and skimmed milk) from the PBS- and DHEA-treated rats, respectively. To ensure that recipients received similar volumes of microbes from the FMT donors, microbial loads in both groups of donors were measured and shown in combination in Additional file 3: Figure S3a. During the 27 days of washout and the 14 days of transplantation, the body weight was measured weekly and was found similar between groups (Fig. 2b). No differences in FBG were observed between groups (Fig. 2c). Compared with rats in the t-PBS group, the t-DHEA group displayed increased glucose levels and area under the curve in glucose tolerance tests (GTT) (Fig. 2d, e). These results show that FMT from PCOS-like rats could lead to glucose intolerance in pseudo germ-free rats. Rats receiving faecal microbiota from PBS-treated donors showed normal oestrous cycles, while their counterparts receiving microbiota from DHEA-treated donors exhibited disrupted oestrous cycles (Fig. 2f). The ovaries of t-PBS rats showed follicles at different stages of development and CL. Rats in the t-DHEA group exhibited several cyst-like follicles with attenuated granulosa cell layers and thickened follicle walls (Fig. 2g). Therefore, faecal microbiota from PCOS-like rats disrupts the oestrous cycle and leads to ovarian morphological anomalies in recipients. The TT, SHBG and FAI levels were higher in the t-DHEA group than in the t-PBS group (Fig. 2h–j). The LH levels decreased insignificantly, while the FSH levels were lower in the t-DHEA group than in t-PBS group (Fig. 2k, l). The LH/FSH ratio was unchanged (Fig. 2m). Interestingly, gut dysbiosis in the DHEA-induced PCOS-like rats led to hyperandrogenism in FMT-recipient rats. In general, FMT from the DHEA group induced impaired glucose tolerance, disrupted oestrous cycle, polycystic ovaries and unbalanced reproductive hormones in recipient pseudo germ-free rats, suggesting an important role of gut dysbiosis in the development of PCOS.

In the DHEA-treated donors, free circulating DHEA was transformed into dehydroepiandrosterone sulfate (DHEA-S) by sulfation in the liver and excreted into the gastrointestinal tract. Untargeted metabolomic analysis showed that DHEA was not detected in the faecal samples of either group; however, the DHEA-S levels were elevated in the DHEA-treated donors (Additional file 3: Figure S3b). Elevated faecal DHEA-S levels were also found in PCOS patients [22]. The DHEA-S levels in faecal samples of both groups were quantified (Additional file 3: Figure S3c). Although there was a significant increase in the DHEA-S level in the gut content of DHEA compared with PBS-treated donors, absolute quantification showed that the levels were insignificant in both groups. The DHEA-S levels were 665 ± 63.21 ng/gram of feces in DHEA-treated donors and 109.8 ± 10.11 ng in PBS-treated donors, which are far below the minimal effecting concentration for elicit a PCOS phenotype [23].

### Gut microbiome and metabolome were changed in FMT-recipient rats

**Fig. 3:**
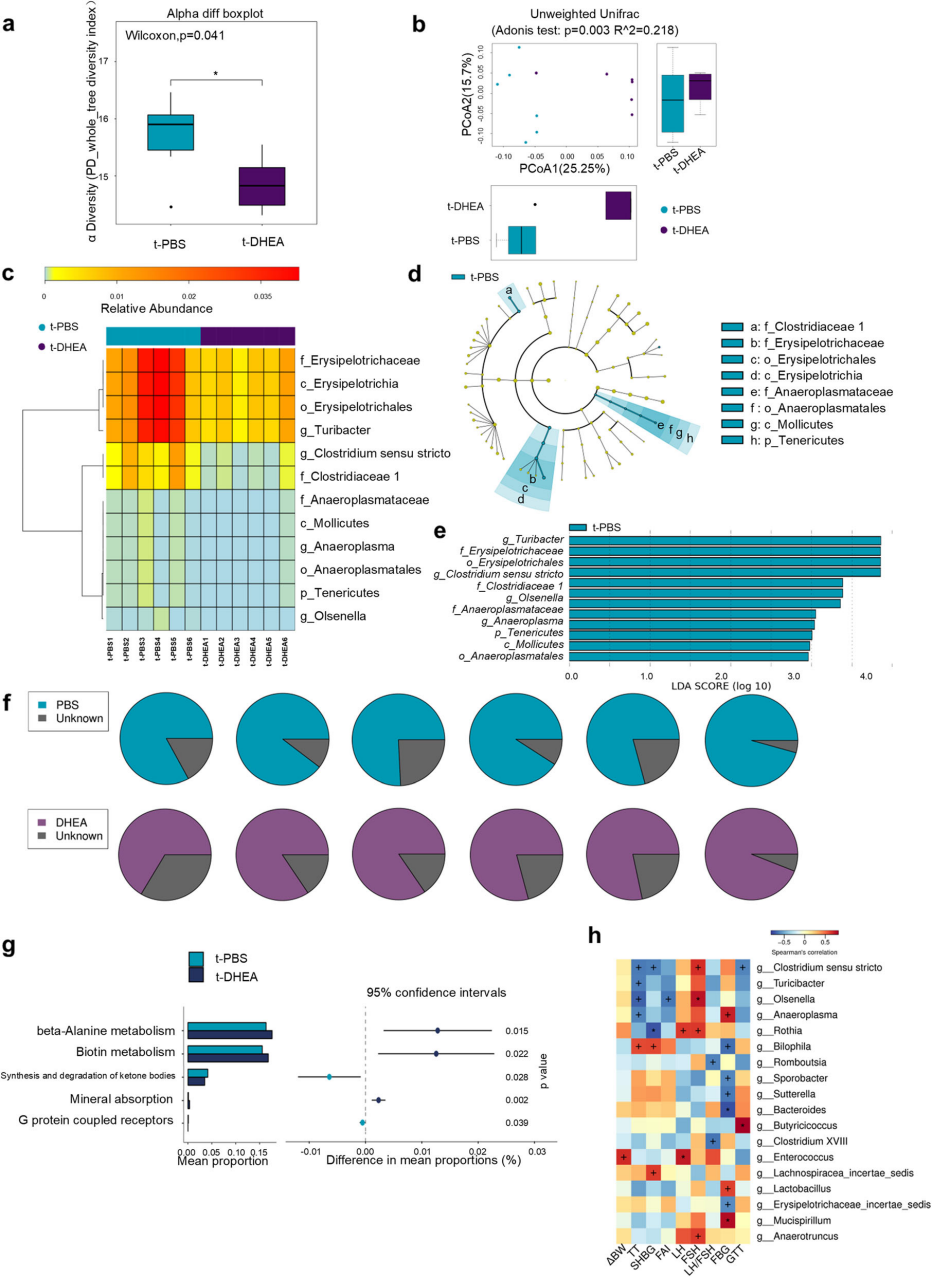
Gut microbiota profiling of FMT recipient rats. **a** Phylogenic diversity whole tree index indicating alpha diversity in the 2 groups. **b** PCoA analysis with unweighted UniFrac distance. Numbers on the axes represent percentages of variation interpreted by that coordinate. **c** Wilcoxon test identified differentially abundant taxa in gut microbiota after being transferred from PCOS-like rats and controls. Cladogram (**d**) and bar plot (**e**) of LEfSe analysis showed taxa contribution to group separation. Taxa with LDA score > 2 were displayed. **f** Pie chart of SourceTracker analysis revealing percentages of gut microbiota originated from their corresponding donors. **g** Differentially enriched modules visualised by STAMP. **h** Heat map of the Spearman’s rho correlation test presenting the association between genus-level microbes and clinical parameters. Red squares represent positive correlations between relative abundances of microbes and clinical parameters; blue squares represent negative correlations. +, *p* < 0.10; *, *p* < 0.05. *n* = 6 rats per group

Faecal samples of the t-PBS and t-DHEA groups were sequenced to confirm the efficiency of FMT and further investigate the underlying mechanisms of FMT induction of the PCOS-like phenotype. Unsurprisingly, the microbial characteristics of DHEA-treated donors reappeared in recipients of FMT from the t-DHEA group. Recipient rats showed a similar alpha diversity index to donor rats (Fig. 3a). Beta diversity analysis revealed that samples from the t-DHEA group formed a distinct cluster from the t-PBS group (Fig. 3b). At the phylum level, *Firmicutes*, *Bacteroidetes*, *Verrucomicrobia* and *Proteobacteria* were most abundant in both the donor and recipient groups (Additional file 4: Figure S4a, d). *Clostridia*, *Bacteroidia*, *Verrucomicrobiae* and *Bacilli* dominated the gut microbiota at the class level in the PBS- and DHEA-treated groups and their recipients (Additional file 4: Figure S4b, e). *Akkermansia*, *Bacteroides*, *Lactobacillus* and *Parabacteroides* ranked most abundant at the genus level before and after FMT in donors and recipients, respectively (Additional file 4: Figure S4c, f). These differentially abundant taxa were overrepresented in the t-PBS group (Fig. 3c), including the genera *Turicibacter* and *Clostridium sensu stricto*, families *Erysipelotrichaceae* and *Clostridiaceae 1*, order *Erysipelotrichales* and class *Erysipelotrichia* which were also increased in PBS-treated donors. Among these differentially abundant microbes, the genus *Turicibacter,* family *Erysipelotrichaceae,* order *Erysipelotrichales* and class *Erysipelotrichia* were top-ranking by LDA score in the LEfSe analysis, which coincided with the gut microbiota profiling of donor rats (Fig. 3d, e). SourceTracker analysis (SourceTracker software version 1.0.1) showed that the donors were the principal origin of t-PBS and t-DHEA rat-gut microbiota (Fig. 3f). Thus, the DHEA-shaped gut microbiota was successfully transferred to recipient rats.

Kyoto Encyclopaedia of Genes and Genomes (KEGG) analysis was performed using the predicted gene abundance to understand the functional contribution of gut dysbiosis. For module enrichment, the synthesis and degradation of ketone bodies and G protein-coupled receptors were enriched in the t-PBS group, while beta-alanine metabolism, biotin metabolism and mineral absorption were enriched in the t-DHEA group (Fig. 3g). These data suggest that aberrant functions in environment perception, energy, vitamin, amino acid and mineral metabolism may occur in the gut microbiota after DHEA-shaped microbiome transplantation. Then, the correlations of genus-level taxa with clinical parameters were assessed (Fig. 3h). The testosterone level was negatively correlated with the relative abundances of *Clostridium sensu stricto*, *Turicibacter*, *Olsenella* and *Anaeroplasma*. The FAI was negatively associated with *Olsenella* levels only. The FSH level was positively associated with *Clostridium sensu stricto*, *Olsenella* and other taxa. In addition, the area under the curve of the GTT, and FBG levels were linked with levels of *Clostridium sensu stricto*, *Anaeroplasma*, and other taxa.

**Fig. 4:**
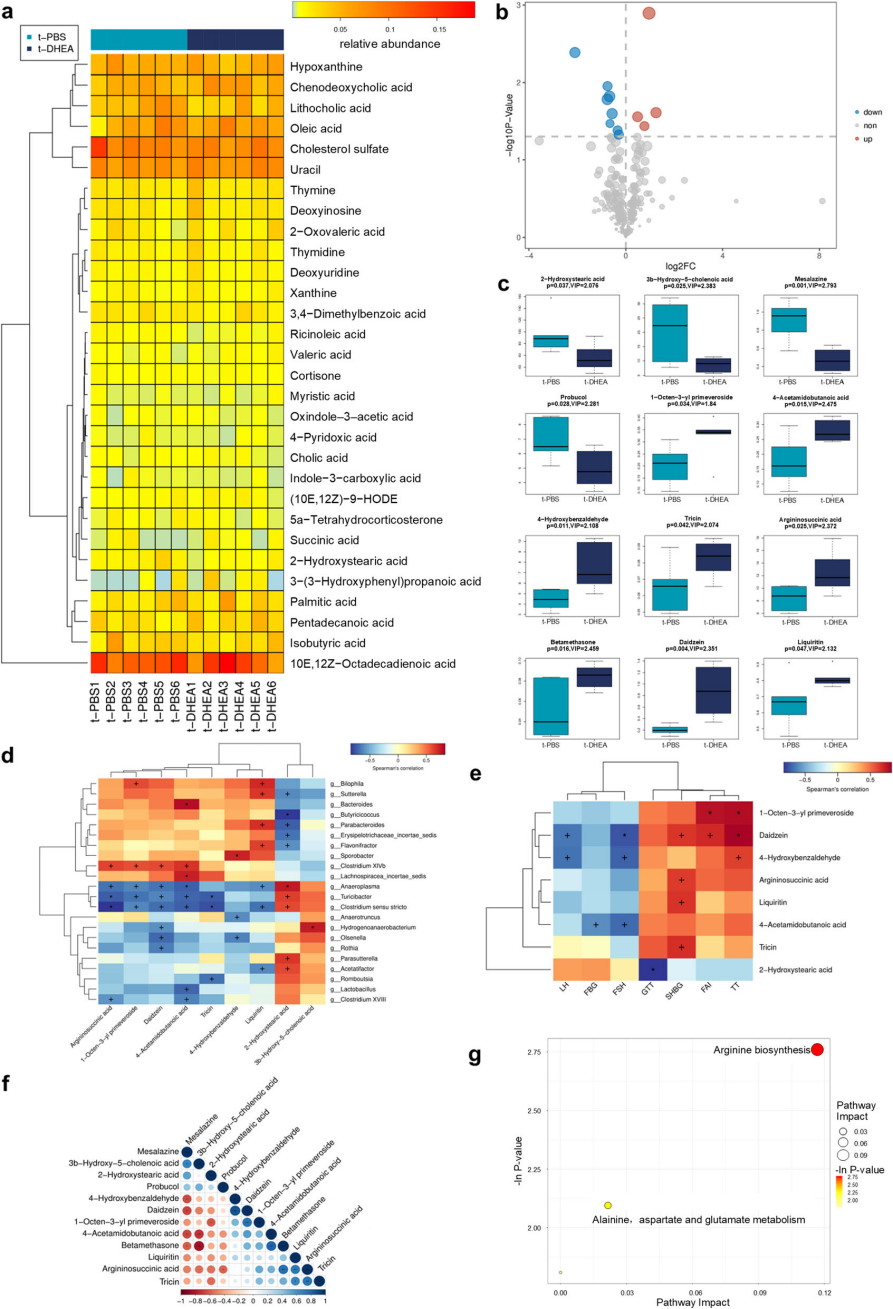
Non-target metabolomic analysis of faecal samples in FMT recipient rats. **a** Heat map presenting top 30 metabolites detected in faecal samples of t-PBS group and t-DHEA group. **b** Volcano plot of the differentially expressed metabolites. **c** Boxplot of the upregulated and downregulated metabolites in t-DHEA group. *p* value of student’s *t* test and variable importance in the projection score of OPLS-DA were both used to select differentially expressed metabolites. Correlation analysis between metabolites and gut microbes (**d**), between metabolites and clinical parameters (**e**) and between each 2 of the differentially expressed metabolites (**f**). Correlations were determined by Spearman’s rho correlation test or Pearson’s correlation test (+ *p* < 0.10; *p* < 0.05). **g** Bubble plot shows differentially enriched metabolic pathways following distinct gut microbiota transplantation. Each bubble indicates an enriched pathway. Horizontal axis and size of the bubble indicate the impact of the pathway. Vertical axis and colour of the bubble indicate the significance of enrichment. *n* = 6 rats per group

The faecal metabolome was investigated to further understand the functional alterations following FMT. The top 30 abundant metabolites are displayed in Fig. 4a. The faecal metabolites with significantly different levels between the t-PBS and t-DHEA groups included amino acid metabolites, fatty acids and bile acids (Fig. 4b, c). The levels of 2-hydroxystearic acid, argininosuccinic acid, 1-octen-3-yl primeveroside, daidzein and 4-acetamidobutanoic acid were significantly associated with the relative abundances of *Anaeroplasma*, *Turicibacter* and *Clostridium sensu stricto* (Fig. 4d). Figure 4e displays differentially expressed metabolites which were correlated with at least one clinical parameter. The correlation analyses between each 2 of these metabolites are shown in Fig. 4f. Functional enrichment analysis showed that arginine biosynthesis and alanine, aspartate and glutamate metabolism were affected by the DHEA-shaped gut microbiota transplantation (Fig. 4g).

### Metabolic phenotypes and genetic changes were characterised in FMT-recipient rat livers

FMT revealed that gut dysbiosis significantly affected metabolic function. The liver serves as the major site for physiological metabolism. Therefore, hepatic genetic expression and morphology were evaluated.

**Fig. 5:**
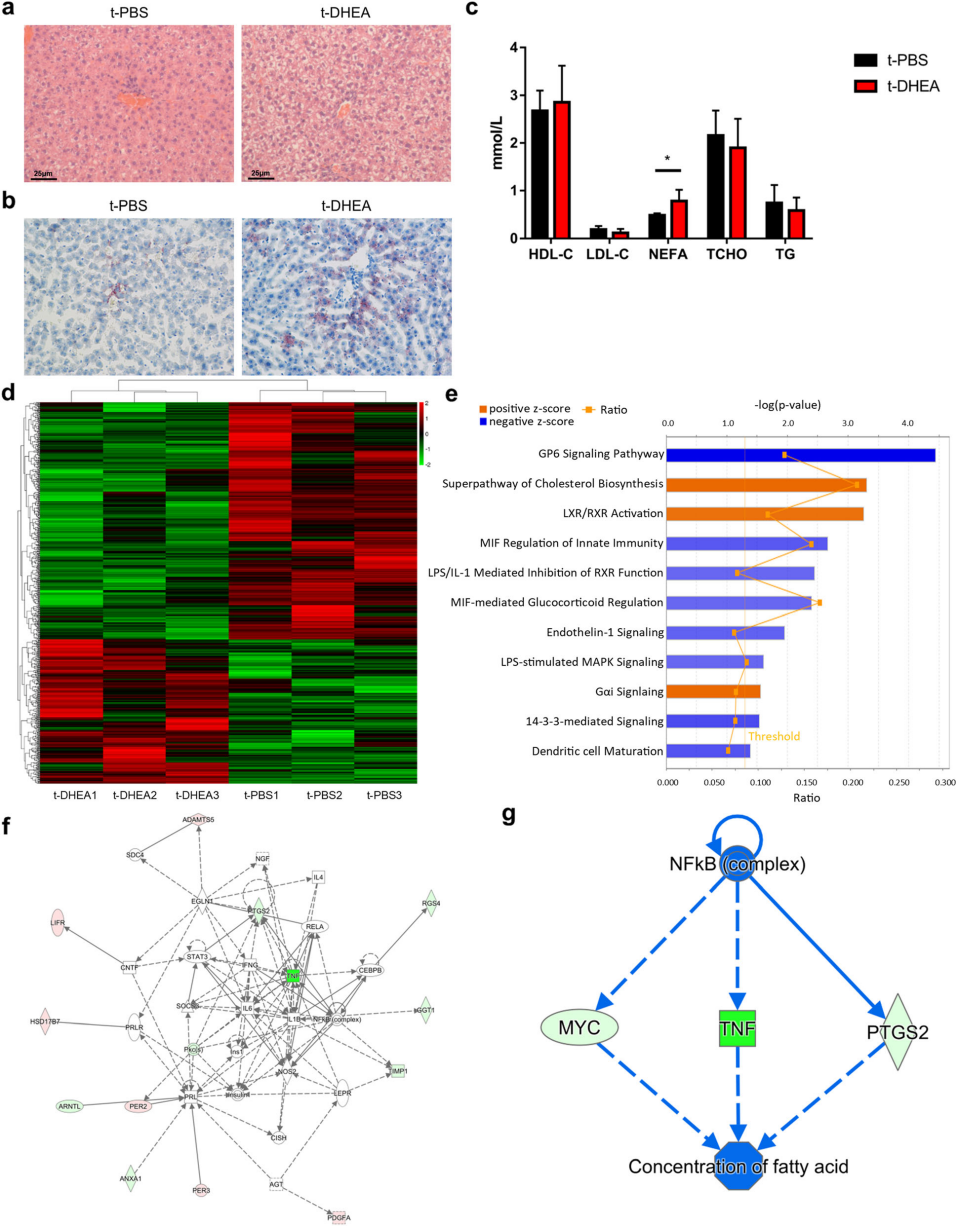
FMT of DHEA-shaped gut microbiota regulated liver metabolism. Representative photomicrographs revealing hepatic lipid content by H&E staining (**a**) and ORO staining (**b**). Scale bar = 25 μm. **c** Serum lipid levels including HDL-C, LDL-C, NEFA, TCHO, and TG in t-PBS group and t-DHEA group (*n* = 6 each group, * *p* < 0.05). Data are given as mean ± SEM. **d** Heat map of 725 DEGs in livers from t-PBS and t-DHEA groups (*n* = 3 each group). These data were presented using FPKM values. **e** Bar plot summarises the significantly influenced canonical pathways regarding metabolism and immunity in rat livers (*n* = 3 each group). Pathways with Z-score more than 2 (orange) or less than − 2 (blue) were displayed and were ranked by − log(*p* value). The straight orange line indicates the significance level of *p* = 0.05. The orange fold line indicates the ratio of enrichment in the pathway. **f** Network presenting interactions between DEGs involved in carbohydrate and lipid metabolism (*n* = 3 each group). Genes are exhibited as red nodes (upregulated) and green nodes (downregulated). Relationships between 2 nodes are indicated as lines (direct interaction) or dotted lines (indirect interaction). **g** Network displaying regulatory effects from upstream regulators to downstream bio-functions linked by DEGs (*n* = 3 each group). *DEGs*, differentially expressed genes; *FPKM*, Fragments Per Kilobase of exon model per million mapped reads; *HDL-C*, high-density lipoprotein cholesterol; *LDL-C*, low-density lipoprotein cholesterol; *NEFA*, non-esterified fatty acid; *TCHO*, total cholesterol; *TG*, triglycerides

Haematoxylin & eosin (H&E) and oil red O (ORO) staining revealed that liver tissue sections in the t-DHEA group contained more and larger lipid droplets than the t-PBS group (Fig. 5a, b). The serum lipid profile was also measured (Fig. 5c). NEFA (non-esterified fatty acids) levels were significantly increased in the t-DHEA group, while high-density lipoprotein cholesterol (HDL-C), low-density lipoprotein cholesterol (LDL-C), total cholesterol (TCHO) and triglycerides (TG) were similar between groups. These results suggest abnormal lipid metabolism; thus, hepatic gene expression profiling was performed to gain more insight into the regulation of glucose and lipid metabolism in the liver. Next-generation sequencing of the liver transcriptome identified differentially expressed genes (DEGs) according to the criteria *p* < 0.05 and fold-change > 1.2. A total of 725 DEGs were selected for subsequent analysis, including 274 upregulated and 451 downregulated genes (Fig. 5d). Canonical pathway analysis using Ingenuity Pathway Analysis (IPA) software revealed that the cholesterol biosynthesis superpathway, LXR/RXR activation and Gαi signalling were upregulated. The downregulated pathways included GP6 signalling, MIF regulation of innate immunity, LPS/IL-1 mediated inhibition of RXR function, MIF-mediated glucocorticoid regulation, endothelin-1 signalling, LPS-stimulated MAPK signalling and 14-3-3-mediated signalling (Fig. 5e). The changes in these pathways indicated that hepatic glucolipid metabolism and immune response were affected by DHEA-shaped gut microbiota transplantation. The interactions between DEGs regarding carbohydrate and lipid metabolism were visualised using IPA (Fig. 5f). Tumour necrosis factor and prostaglandin-endoperoxide synthase-2 (PTGS2) occupied the centre of the working network. Regulator effects analysis was used to combine upstream regulator analysis and downstream function analysis (Fig. 5g). It indicated that alterations in hepatic gene expression regulated fatty acid concentration, which coincides with dyslipidaemia. These data indicated that the dysregulation of hepatic gene expression might be involved in metabolic derangement triggered by the DHEA-shaped microbiome transplantation.

## Discussion

Although PCOS is a serious health concern worldwide, the limited treatment options primarily focus on the symptoms and therefore yield unsatisfactory therapeutic outcomes and long-term metabolic complications, primarily due to a lack of understanding of its aetiology. Gut dysbiosis is characteristic of PCOS patients and rodent models. The manipulation of gut microbiota by FMT, probiotics, prebiotics or symbiotics ameliorates the PCOS phenotype [20, 24]. Recent studies have revealed that gut microbiota shows a close relationship with normal sex hormone levels in rodent models; Yurkovetskiy et al. found that the microbiota of castrated male mice was closer to female mice compared with uncastrated male counterparts, verifying that androgens affect gut microbiota [7]. FMT from males increased the serum T level in immature female recipients, which suggests that gut microbes regulated serum T levels [8]. Therefore, there is a mutual interaction between androgens and the microbial community. However, whether the bidirectional relationship between androgens and the microbiome participates in the development of PCOS is not known. Consequently, the precise role of gut dysbiosis in the pathogenesis of hyperandrogenism-dependent metabolic disorders is worthy of further investigation.

The findings that androgens can induce PCOS phenotypes and gut microbiota disturbance in rodents [19, 25] were validated in DHEA-induced PCOS-like rats. Clinical studies found that the gut microbial composition and relative abundance of taxa were related to PCOS phenotypes [18]. However, it is not clear whether DHEA leads to the PCOS metabolic and endocrinal phenotypes directly or if this process depends on the presence and alteration of the gut microbiota [26]. In this study, antibiotic cocktails were administered to clear the existing gut microbes during model establishment [27]. Zarrinpar et al. found that depletion of the microbiome by antibiotics reduced FBG levels and the GTT glucose surge in normal mice [28]. However, the blood glucose curves showed similarly high levels in the DHEA and DHEA + antibiotics groups, suggesting that hyperandrogenism leads to glucose intolerance independent of the gut microbiome. The clearance of gut microbiota did not avert polycystic morphology induced by DHEA, suggesting that the ovarian anomalies resulting from androgen administration were independent of the gut microbiota. However, the clearance of gut microbiota by ABX decreased serum T level and FAI in the DHEA + antibiotics group. After glucuronidation and sulfation, androgens were excreted by the liver via bile or the kidneys via urine. The glucuronidases and sulfatases synthesised by gut microbes can deconjugate and facilitate the reabsorption of androgens. Decreased serum T levels and FAI may be related to decreased reabsorption and increased excretion of androgens through the gut. The LH and FSH levels and the LH/FSH ratio simultaneously decreased significantly. The relationship between gut microbiota and pituitary gonadotropic hormones has not been elucidated. The concurrent decrease in gonadotropins possibly resulted from suppressed gonadotropin-releasing hormone (GnRH) levels in the hypothalamus. A previous study reported that compared with conventional mice, differentially expressed lncRNAs in the hippocampus of germ-free mice were closely associated with GnRH. This finding indicates that the gut microbiota is implicated in gonadotropin regulation. In general, gut microbiota may not play a role in the development of metabolic and ovarian dysfunctions in DHEA-induced PCOS. However, significant alterations in endocrine hormone levels in pseudo germ-free rats indicate an association between gut microbiota and the hypothalamus–pituitary–endocrine gland axes.

Germ-free and pseudo germ-free animals associated with FMT were used to illustrate the causal relationship between gut microbiota and diseases such as intestinal carcinogenesis and irritable bowel syndrome [29, 30]. The relatively long-term impact of androgen-shaped gut microbiota from female donors on pseudo germ-free recipients was studied for the first time by combining ABX and FMT. In this study, the 21-d antibiotic treatment was divided into 3 courses of 7 days with a 2-day interval to prepare the recipient rats for FMT [31]. The microbiota was transferred to the recipients by oral gavage within 30 min of excretion to maintain bacterial viability. The impact of the DHEA-shaped gut microbiome was analysed and discussed from metabolic and endocrinal perspectives.

In a landmark study, FMT from obese conventional mice to germ-free counterparts caused a significant increase in body weight in the recipients, suggesting an association between the gut microbiome and body weight [32]. In this study, excluding the effect of obesity on metabolism and endocrine functions in FMT recipients, body weight was similar between the t-PBS and t-DHEA groups. Glucose metabolism was closely correlated to the gut microbiota, and dietary fibre-induced gut microbiota relieved T2DM [11, 33]. Although the baseline blood glucose was similar between groups, rats in the t-DHEA group showed significantly elevated blood glucose levels and area under the curve in GTT. Compared with the t-PBS group, a decreased relative abundance of *Turicibacter* and *Clostridium sensu stricto* in gut microbiota was observed in the t-DHEA group. Previous studies showed an association of these genera with glucose metabolism. The decreased relative abundance of *Turicibacter* was negatively correlated with impaired glucose tolerance induced by rapamycin treatment [34]. *Clostridium sensu stricto* and its family *Clostridiaceae* were regarded as biomarkers of previous gestational diabetes mellitus [35]. Therefore, the glucose intolerance in recipients of DHEA-shaped microbiomes may result from dysbiosis. Glucose intolerance and increased circulating NEFA levels were both regarded as early markers for metabolic derangement and insulin resistance [36]. NEFA administration damaged mitochondrial function and increased reactive oxygen species production, thus inducing hepatic lipid accumulation and insulin resistance [37]. In this study, rats in the t-DHEA group displayed elevated serum NEFA levels and hepatic steatosis, indicating metabolic derangement and potential insulin resistance. An analysis of the functional enrichment of the faecal metabolome indicated arginine biosynthesis alteration. Arginine was associated with glucose and lipid metabolism, and dietary supplementation with arginine was beneficial to metabolic syndrome, including obesity, diabetes and dyslipidaemia [38]. A recent meta-analysis validated the impact of arginine supplementation on the lipid profile [39]. Since arginine biosynthesis alteration was triggered by the FMT of DHEA-shaped gut microbiota, androgens may regulate glucolipid metabolism through dysregulated arginine biosynthesis. In addition, the arginine level served as an early biochemical marker of underlying cardiovascular complications in PCOS [40]. It was reported that gut microbes could distally control hepatic gene expression, resulting in transcriptional variants of hepatic metabolism [41]. The liver transcriptome was used to determine the effect of changes in gut microbes and microbial metabolites on glucose and lipid metabolism. The effect of transferred gut microbiota on recipient liver gene expression was not significant; however, there were some changes. Therefore, a 1.2-fold-change was employed to analyse the underlying variations [42, 43]. The effects of DHEA-shaped gut microbiota on gene transcription in the liver have rarely been studied. Hepatic gene expression profiling showed that DHEA-shaped gut microbiota perturbed glucose metabolism, cholesterol metabolism and immune response, which was in accordance with the glucose intolerance, liver lipid accumulation and dyslipidaemia exhibited in PCOS-like rat-FMT recipients. Thus, the gut microbiota in DHEA-treated rats possibly disrupted glucose and lipid metabolism by regulating hepatic gene expression.

There are mutual interactions between androgen and gut microbiota [7, 8]. Nevertheless, it has not been determined whether there is a bidirectional effect between hyperandrogenism and gut dysbiosis or how it might participate in the development of PCOS. DHEA-induced gut dysbiosis was confirmed in the rat PCOS model. Furthermore, FMT from DHEA-induced PCOS-like rats increased serum T level in recipients, indicating the regulatory effect of the gut microbiome on serum T levels (Additional file 5: Figure S5). DHEA-S in the transplant and gut microbiota were simultaneously transferred to recipients. There is a common belief that DHEA and DHEA-S can interconvert freely. However, a previous study found no change in serum DHEA levels after oral administration of DHEA-S and concluded that DHEA-S is not the origin of bioavailable DHEA [44]. As DHEA was not detected and DHEA-S levels were very low in the transplant, we considered that the effect of FMT in the transplant may be irrelevant to either DHEA or DHEA-S and was mainly attributed to the transfer of gut microbiota. There are several potential mechanisms by which the gut microbiome influences serum T levels. Markle et al. reported that elevated T level was attributed to increased hormone production due to the increased androgen precursor level [45]. Both ovary and adrenal glands could be the origins of these androgens. The adrenal gland is the effector organ of the hypothalamus–pituitary–adrenal gland (HPA) axis. Gut dysbiosis disturbs the homeostasis of the HPA axis, which is essential in coping with stress [46]. Stressed animal models displayed declined *Turicibacter* and *Clostridium sensu stricto*, suggesting an association between stress and the reduced relative abundance of *Turicibacter* and *Clostridium sensu stricto* [47]. These 2 genera were depleted in the t-DHEA group, indicating that the HPA axis function was possibly altered in these rats. The dysregulated HPA axis may perturb androgen production and T levels [48]. On the other hand, dysbiosis of gut microbiota can lead to metabolic derangement, including insulin resistance [49, 50]. The subsequent hyperinsulinemia causes excessive androgen production in the ovaries and increases circulating T. Nevertheless, FMT-induced hyperandrogenism may be a vital factor in ovarian morphological anomalies and disrupted oestrous cycles. This phenomenon deserves further investigation owing to its promising roles in the development and evolution of PCOS. A derangement of gonadotropin levels occurred in FMT recipients of PCOS-like rats. The LH and FSH levels decreased in the t-DHEA group and were associated with gut microbes. Together with hyperandrogenism, suppressed serum FSH levels contributed to deteriorating ovarian morphology and oestrous cycles and reduced levels of gonadotrophins implied GnRH suppression which was possibly regulated by the gut–brain axis. The gut–brain axis refers to the mutual communication between the gut and the central nervous system [51]. The hypothalamus is a primary location in the brain responsible for receiving nervous and humoral regulation signals from the gut microbiome. De Palma et al. showed that variations in the gut microbiota could influence the functioning of the hypothalamus [46]. In addition, the hypothalamus is also the upstream commander of many endocrine axes including the hypothalamus–pituitary–ovary (HPO) axis. It is known that dysregulation in the HPA axis inhibits the HPO axis. Therefore, decreased GnRH and gonadotropins may be attributed to potential HPA dysfunction. In addition, arginine levels regulate GnRH synthesis and secretion. Serum arginine levels were reduced in PCOS patients, and it was found that L-arginine administration could ameliorate hormonal disturbances in PCOS rats [52]. Besides, arginine is the precursor of nitric oxide, which plays a critical role in the neuroendocrine regulation of reproduction and is involved in controlling the activity of GnRH neurons [53]. Therefore, GnRH secretion may be affected by gut microbiota dysbiosis and metabolome shifts, thereby disturbing the HPO axis.

Overall, gut microbiota from PCOS-like hyperandrogenic rats induces glucose and lipid metabolism disorder, disrupted oestrous cycles, polycystic ovaries and reproductive hormone variations in pseudo germ-free recipients. Therefore, gut dysbiosis may coordinate with hyperandrogenism to disrupt metabolic and endocrinal homeostasis through disturbance of the HPA and HPO axes. In PCOS, this process is potentially regulated by the gut–brain and gut–liver axes (Additional file 5: Figure S5).

## Conclusions

DHEA treatment induces the PCOS-like phenotype and gut dysbiosis in SD rats. The establishment of the PCOS model in pseudo germ-free rats shows that gut microbiota depletion cannot prevent PCOS-like phenotypes in DHEA-treated rats and hyperandrogenism remains the primary factor in PCOS pathogenesis. FMT-oriented gut dysbiosis causes PCOS-like phenotypes, including metabolic and endocrinal malfunction in recipients. Therefore, androgen-shaped gut microbiota also plays a part in PCOS pathogenesis. These novel findings shed light on the understanding of new etiological agents and potential comprehensive PCOS treatment options.

## Methods

### Animals

Thirty SD rats (female, 3-week old) were purchased from Vital River Laboratory Animal Technology Co Ltd., Beijing, China. Two rats per cage were housed in 15 individually ventilated cages in a specific pathogen-free environment and maintained under a 12-h light/dark cycle, controlled temperature and stable humidity, with 24 h free access to standard irradiated rodent feed and non-acidified drinking water. The rats’ body weights were measured weekly.

### Establishment of PCOS model

Eighteen rats were randomly allocated to 3 groups: PBS, DHEA and DHEA + antibiotics groups (*n* = 6 each group). To induce the PCOS phenotype, rats in the DHEA group were subcutaneously injected with 6 mg/100 g bodyweight DHEA (cat. no. H10940064, Yangzhou Pharmaceutical Co., Ltd) daily for 35 consecutive days. Rats in the PBS group were injected with PBS. Rats in the DHEA + antibiotics group were injected with DHEA in the same manner as the DHEA group and received a freshly prepared antibiotic cocktail daily in their drinking water. The antibiotic cocktail, consisting of ampicillin sodium (1 mg mL^−1^), neomycin sulfate (1 mg mL^−1^), metronidazole (1 mg mL^−1^), and vancomycin hydrochloride (0.5 mg mL^−1^) (all purchased from Sangon Biotech, China) [54], was administered to rats every day immediately after preparation. The rats in all 3 groups were sacrificed after 5 weeks of treatment.

### Establishment of FMT model

Twelve other rats were randomly allocated into 2 groups, a t-PBS and a t-DHEA group (*n* = 6 each group). First, a three-course antibiotic treatment was administered to both groups of FMT recipient rats to establish the pseudo germ-free rat model as previously described [31, 54]. They were given an antibiotic cocktail in drinking water for 7 days, non-acidified drinking water for 2 days, the antibiotic cocktail in drinking water for 7 days, non-acidified drinking water for 2 days, the antibiotic cocktail in drinking water for 7 days, and non-acidified drinking water for 2 days before FMT. The faecal samples were collected and processed within 30 min to ensure bacterial viability in the transplant. The faecal pellets were pooled and homogenised with 1 mL PBS and 1 mL skimmed milk per pellet (0.2 g) [21]. Then, the mixture was allowed to settle by gravity for 5 min, and 1 mL supernatant was transferred to recipients by oral gavage every day for 14 days. After the 21-day PCOS model establishment, the donors continued to receive DHEA for another 14 days during FMT. The microbial loads in donors after DHEA treatment and in recipients after ABX treatment were assessed using MAKLER counting chamber. The recipients received approximately 1 × 10^9^ microbes by oral gavage daily and were sacrificed after 2 weeks of FMT.

### GTT

All rats were fasted overnight for 16 h before GTT. The blood glucose levels were measured using tail vein blood before and 15, 30, 45, 60, 90 and 120 min after intraperitoneal glucose administration (50% glucose, 2 g/kg body weight). The total area under the GTT curve was calculated using GraphPad Prism 7.00.

### Vaginal smears and oestrous cycle determination

Vaginal smears were taken daily between 9:00 am and 11:00 am for 8 consecutive days before sacrifice. The oestrous cycle stage was determined by observation of the vaginal smears using a light microscopic (Zeiss, Germany). Briefly, samples dominated by nucleated epithelial cells indicated the proestrus stage, and those with primarily cornified squamous epithelial cells indicated the estrus stage. The metestrus stage was indicated by comparative quantities of cornified squamous epithelial cells and leukocytes, while the diestrus stage was dominated by leukocytes.

### Organ collection and histology

The rats were euthanised by cervical dislocation. The ovaries were carefully dissected and fixed in 4% paraformaldehyde overnight at room temperature before being embedded in paraffin. The livers were also carefully separated. A sample of liver tissue was snap-frozen in liquid nitrogen and kept at – 80 °C for future use. Others were fixed and embedded using the same procedure as the ovaries. Eight-micrometre-thick tissue sections of ovaries and livers were processed by H&E and ORO staining according to standard histological procedures and examined using a light microscope (Zeiss, Germany).

### Serum analysis

At the end of the treatments, blood samples were collected from the abdominal aorta of anaesthetised rats using a pro-coagulation tube and centrifuged at 2500 rpm for 15 min at 4 °C. Then the upper supernatants were carefully transferred to cryogenic vials and immediately stored at – 80 °C for further evaluation. The levels of TT (582701, Cayman, USA), SHBG (MBS014745, Mybiosource, USA), LH (MBS2018978, MyBioSource, USA) and FSH (EKU04249, BIOMATIK, Canada) were analysed using enzyme-linked immunosorbent assay kits. To assess the biologically active amount of testosterone, the FAI was calculated using the following formula: FAI = (TT × 100)/SHBG [55]. Separate serum samples were sent to the Shanghai Model Organisms Center, Inc., to evaluate serum lipids.

### Faecal sample collection and DNA extraction

The faecal pellets were collected in cryogenic vials immediately after being discharged before sacrifice. The faecal samples were snap-frozen and stored at – 80 °C before analysis. Microbial DNA was extracted from rat faecal samples using QIAamp Fast DNA Stool Mini Kit (51604, Qiagen, Hilden, Germany) according to the manufacturer’s instructions. A Class II biosafety cabinet was used for the preparation of the extracted DNA. The studies were carried out attentively by the same technician to reduce the risks of contamination or operational mistakes. The quantity and purity of the extracted DNA were controlled using NanoDrop 2000 (Thermo Scientific, USA). The integrity and size of the isolated DNA were evaluated using agarose gel electrophoresis.

### 16S rRNA gene amplification and sequencing

The V3–V4 hypervariable region of the 16S rRNA gene was amplified by PCR under the following conditions: 95 °C for 3 min, followed by 30 cycles at 98 °C for 20 s, 58 °C for 15 s, 72 °C for 20 s and a final extension at 72 °C for 5 min. The primers used were 341F 5′-CCTACGGGRSGCAGCAG)-3′ and 806R 5′-GGACTACVVGGGTATCTAATC-3′ [56]. The 30-μL reaction system consisted of 15-μL 2 × KAPA Library Amplification ReadyMix, 20-μM primers, 50-ng template DNA and double-distilled water. The amplification products were isolated using 2% agarose gel, purified using a AxyPrep DNA Gel Extraction Kit (Axygen Biosciences, USA), and quantitatively measured by Qubit®2.0 (Invitrogen, USA). After library establishment, the tags were sequenced using the MiSeq platform (Illumina, Inc., USA) for 250-bp paired-end reads.

### Process of 16S sequencing data

After trimming the primers and barcodes, the lengths and average base quality of the tags were evaluated. The 16S tags were limited to the region between 220 bp and 500 bp. The average Phred score of the bases were at least 20 (Q20), and at most 3 ambiguous N. The enumeration of the tags was followed by the removal of redundancy. Only the reliable tags (frequency > 1) were allocated into operational taxonomic units (OTUs) labelled by a representative tag. Next, Userach (version 7.0) was used to cluster the OTUs at 97% similarity and filter out the chimeric sequences. Every representative tag was aligned against the RDP database (http://rdp.cme.msu.edu) and assigned to a taxon (confidence threshold: 0.8). Tags which could not be distributed to any known taxonomy level were defined as “unclassified.” Python scripts of Qiime (V1.9.1) were included for OTU standardisation, OTU profiling and alpha/beta diversity assessment. Random levelling of samples was performed to reduce the deviation resulting from size differences. The microbial richness and evenness were evaluated by α-diversity by the PD whole tree index, which is based on the phylogenetic tree and evaluates diversity by summing the length of all branches. PCoA, adonis analysis and ANOSIM were employed to differentiate the microbial composition between groups [57]. The non-parametric Wilcoxon test was used to determine the statistical significance of taxa and KEGG orthologues (KOs). The differences in the relative abundance of bacterial taxa between groups were identified using the LEfSe method (version 1.0). The significance level was set at *p* < 0.05 and the effect size at LDA score > 2.0. STAMP (version 2.1.3) was used to identify differentially enriched KO modules between groups. Correlation analysis between gut microbiota and clinical parameters, gut microbiota and faecal metabolome, and faecal metabolome and clinical parameters was conducted using Spearman’s rho correlation test. The degree of transfer of gut microbiota from donor rats to pseudo germ-free rats was evaluated using SourceTracker software version 1.0.1.

### Gut microbiota metabolite analysis

Faecal metabolome analysis was conducted using Liquid Chromatography-Mass Spectrometry (LC-MS, Agilent 1290 UHPLC). The data were collected using AB 5600 Triple TOF under the control of Analyst TF 1.7 (AB Sciex). The raw data were converted using ProteoWizard and processed using XCMS, followed by metabolite identification. The metabolites were annotated using m/z, retention time, and MS/MS fragmentation patterns. The differential metabolites between groups were identified using the student’s *t* test and orthogonal projections to latent structures-discriminant analysis (OPLS-DA). Volcano and bubble plots were produced using the R package (version 3.5.1). The corrplot package was used for correlation analysis, the pheatmap package was used for hierarchical clustering, and the ropls package for OPLS-DA. DHEA-S was quantified in each pellet of the donor animals’ faeces using LCMSMS.

### Library construction for mRNA-seq and sequencing

A RNeasy mini kit (Qiagen, Germany) was used to extract total RNA from the rat livers. The TruSeq® RNA Sample Preparation Kit (Illumina, USA) was employed to construct paired-end libraries based on the manufacturer’s guidelines. An Agilent 2100 bioanalyser was used to evaluate the concentration and size distribution of the cDNA library (Agilent Technologies, USA). Finally, the libraries were sequenced on the HiSeq Xten platform (Illumina, USA).

### Process of mRNA-seq data

First, the raw reads were filtered by Seqtk (https://github.com/lh3/seqtk). Clean reads were obtained after adapter removal, quality control and host contaminant filtering of raw reads using Seqtk. Quality control was based on the Phred score of each base being more than 20, and the read length greater than 25 bp. Hisat2 (version 2.0.4) was employed to map the cleaned reads to Rattus norvegicus Rnor_6.0 [58]. The genes fragments were counted using Stringtie (version:1.3.0) followed by FPKM (Fragments Per Kilobase of exon model per million mapped reads) normalisation [59]. EdgeR was used to identify the DEGs based on FPKM values with the thresholds of *p* < 0.05 and fold-change > 1.2. The heat map was produced by R package pheatmap. IPA software was used for bioinformatics analysis of DEGs.

### Statistics

GraphPad Prism 7.00 was used to perform statistical analyses and data visualisation. The sample sizes were calculated using GraphPad StatMate 2.0 with power analysis. A Kolmogorov–Smirnov test was performed to examine the quantitative data for Gaussian distribution. A two-tailed unpaired student’s *t* test, Mann–Whitney test and one-way analysis of variance coupled with Turkey’s multiple comparisons test were performed to assess the differences between and among groups. The quantitative data were shown as means ± standard error of the mean (SEM). A difference of *p* < 0.05 was considered statistically significant.

## Supplementary Information

**Additional file 1: Figure S1.** Microbial characteristics of DHEA-induced PCOS-like rats.

**Additional file 2: Figure S2.** Depleting the gut microbiota by antibiotic treatment in DHEA+ABX group.

**Additional file 3: Figure S3.** Transplant contents in FMT donors and gut circumstances in antibiotic-treated recipients before FMT initiation.

**Additional file 4: Figure S4.** Microbial composition in FMT donors and recipients.

**Additional file 5: Figure S5.** Effects of DHEA-shaped gut microbiome transplantation in recipient rats.

## Data Availability

The 16S rRNA gene sequencing data have been deposited in the NCBI database under accession code PRJNA635286. Hepatic mRNA sequencing data is available at NCBI Gene Expression Omnibus (GEO), series accession number GSE151220. All data can be obtained in this manuscript or from the authors upon request.

## Abbreviations

PCOS: Polycystic ovary syndrome
DHEA: Dehydroepiandrosterone
DHEA-S: Dehydroepiandrosterone sulfate
T2DM: Type 2 diabetes mellitus
CVD: Cardiovascular disease
FMT: Faecal microbiota transplantation
LH: Luteinising hormone
CL: Corpora lutea
TT: Total testosterone
SHBG: Sex hormone-binding globulin
FAI: Free androgen index
FSH: Follicle-stimulating hormone
ABX: Antibiotics
GTT: Glucose tolerance test
KEGG: Kyoto Encyclopaedia of Genes and Genomes
H&E: Haematoxylin & eosin
ORO: Oil red O
NEFA: Non-esterified fatty acids
HDL-C: High-density lipoprotein cholesterol
LDL-C: Low-density lipoprotein cholesterol
TCHO: Total cholesterol
TG: Triglycerides
DEGs: Differentially expressed genes
IPA: Ingenuity Pathway Analysis
GnRH: Gonadotropin-releasing hormone
HPA: Hypothalamus–pituitary–adrenal gland
HPO: Hypothalamus–pituitary–ovary
SD: Sprague Dawley
OTUs: Operational taxonomic units
PCoA: Principal coordinate analysis
ANOSIM: Analysis of similarities
LDA: Linear discriminant analysis
LEfSe: LDA effect size
KOs: KEGG orthologues
LC-MS: Liquid chromatography–mass spectrometry
OPLS-DA: Orthogonal projections to latent structures-discriminant analysis
FPKM: Fragments Per Kilobase of exon model per Million mapped reads
SEM: Standard error of the mean
