# Supplementary material for: Androgen-induced gut dysbiosis disrupts glucolipid metabolism and endocrinal functions in polycystic ovary syndrome
Source: Microbiome. 2021 May 6;9:101. doi: 10.1186/s40168-021-01046-5 (PMC8103748; doi:10.1186/s40168-021-01046-5)
Supplement: Supplementary file 2 — Additional file 1: Figure S1. Microbial characteristics of DHEA-induced PCOS-like rats. [file 40168_2021_1046_MOESM2_ESM.pdf]

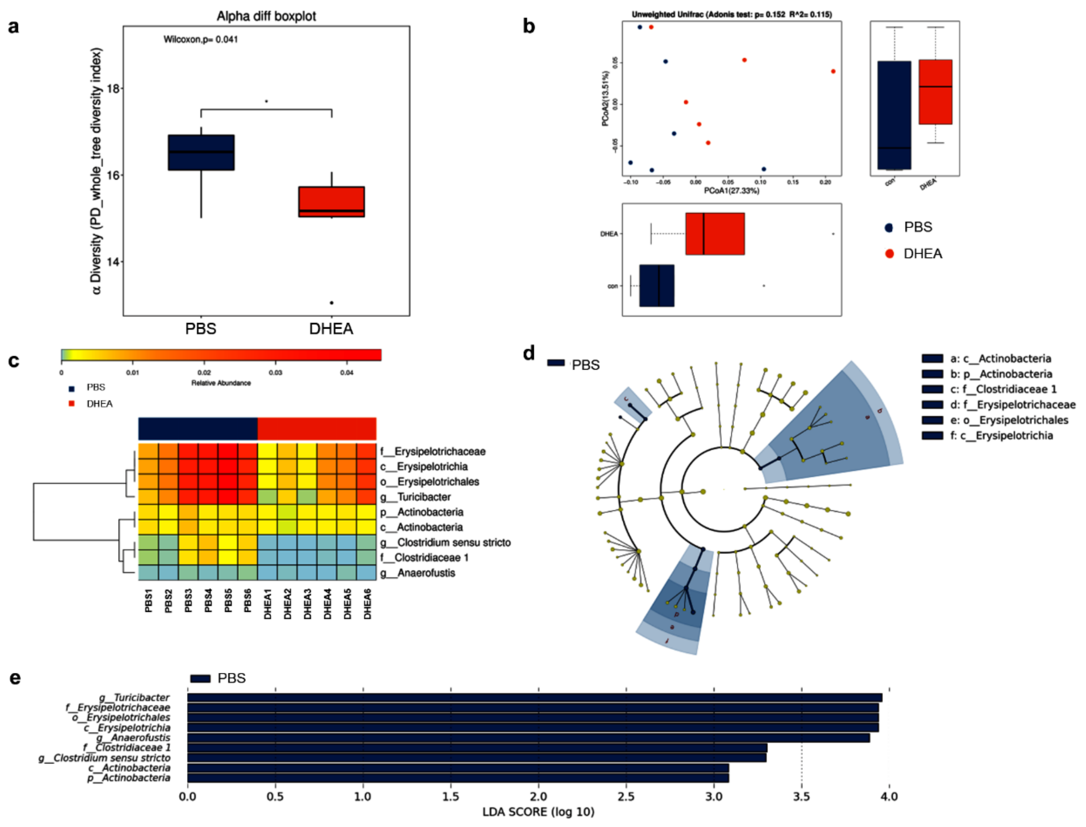

Figure S1. Microbial characteristics of DHEA-induced PCOS-like rats.

a Alpha diversity was presented by PD whole tree index. **b** Beta diversity was displayed by PCoA analysis with unweighted UniFrac distance. Numbers on the axes represent percentage of variation interpreted by that coordinate. **c** Heat map of differentially abundant taxa between samples from DHEA treated rats and controls. These taxa were identified by Wilcoxon test with $p<0.05$. LEfSe analysis results were displayed by cladogram (**d**) and LDA score bar plot (**e**). Differentially abundant taxa enriched in PBS group were represented by blue sectors or bars. Taxa with LDA score >2 were shown and regarded capable of discriminating groups. n=6 rats each group.
