# Supplementary material for: Androgen-induced gut dysbiosis disrupts glucolipid metabolism and endocrinal functions in polycystic ovary syndrome
Source: Microbiome. 2021 May 6;9:101. doi: 10.1186/s40168-021-01046-5 (PMC8103748; doi:10.1186/s40168-021-01046-5)
Supplement: Supplementary file 3 — Additional file 2: Figure S2. Depleting the gut microbiota by antibiotic treatment in DHEA+ABX group. [file 40168_2021_1046_MOESM3_ESM.pdf]

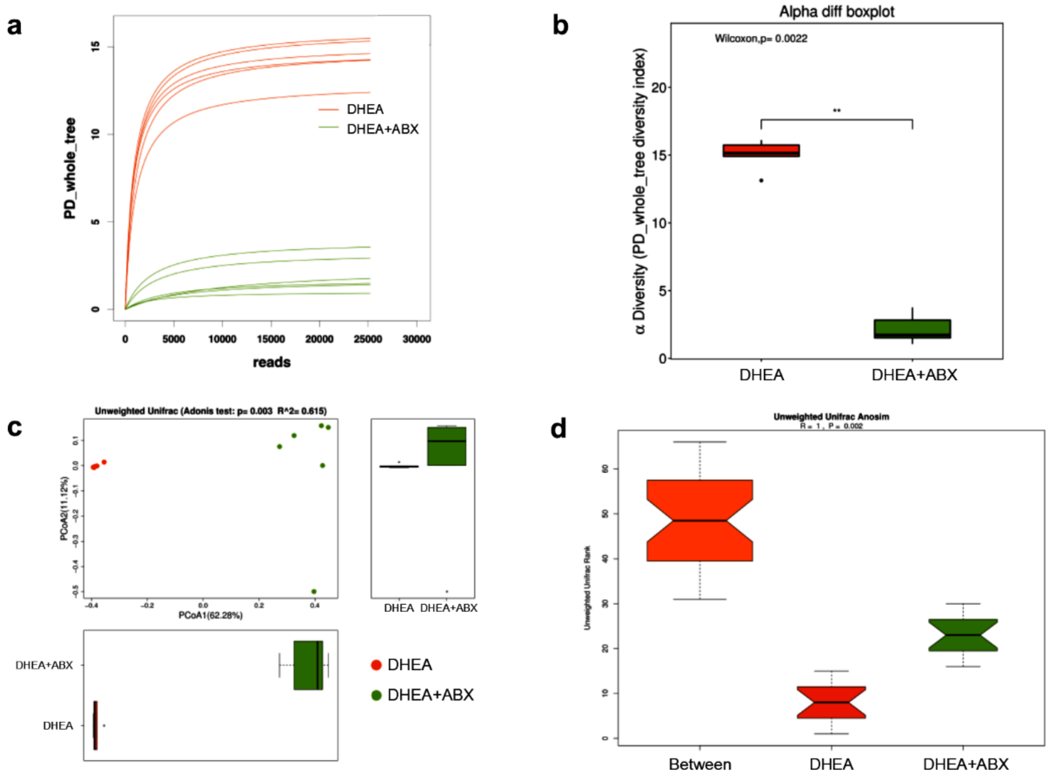

Figure S2. Depleting the gut microbiota by antibiotic treatment in DHEA+ABX group.

a Melting curves of 12 fecal samples in DHEA group and DHEA+ABX group. **b** PD whole tree index indicating alpha diversity of microbiomes in the two groups. PCoA analysis (**c**) and ANOSIM analysis (**d**) performed using unweighted UniFrac distance to assess beta diversity between microbial communities of the two groups. DHEA+ABX, DHEA+antibiotics. n=6 each group.
