# Supplementary material for: Androgen-induced gut dysbiosis disrupts glucolipid metabolism and endocrinal functions in polycystic ovary syndrome
Source: Microbiome. 2021 May 6;9:101. doi: 10.1186/s40168-021-01046-5 (PMC8103748; doi:10.1186/s40168-021-01046-5)
Supplement: Supplementary file 5 — Additional file 4: Figure S4. Microbial composition in FMT donors and recipients. [file 40168_2021_1046_MOESM5_ESM.pdf]

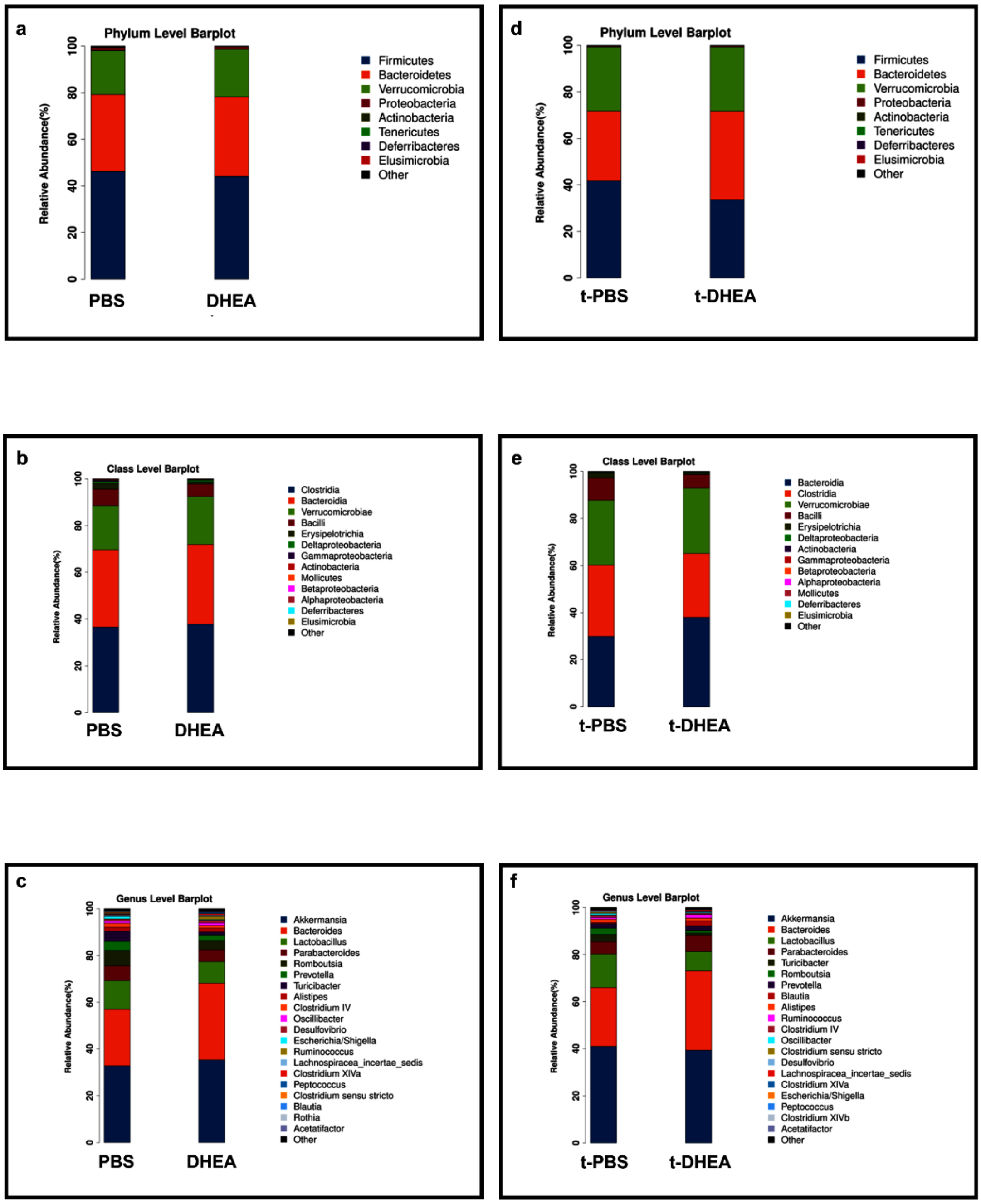

Figure S4. Microbial composition in FMT donors and recipients.

Bar plot of phylogenetic composition of phyla-level (a), class-level (b), and genus-level (c) bacterial taxa in PBS-treated rats and DHEA-treated rats.

Bar plot of phylogenetic composition of phyla-level (d), class-level (e), and genus-level (f) bacterial taxa in FMT recipients.
