# Supplementary material for: Androgen-induced gut dysbiosis disrupts glucolipid metabolism and endocrinal functions in polycystic ovary syndrome
Source: Microbiome. 2021 May 6;9:101. doi: 10.1186/s40168-021-01046-5 (PMC8103748; doi:10.1186/s40168-021-01046-5)
Supplement: Supplementary file 4 — Additional file 3: Figure S3. Transplant contents in FMT donors and gut circumstances in antibiotic-treated recipients before FMT initiation. [file 40168_2021_1046_MOESM4_ESM.pdf]

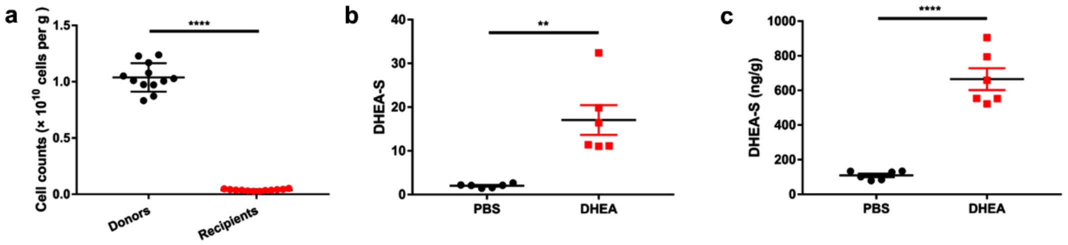

Figure S3. Transplant contents in FMT donors and gut circumstances in antibiotic-treated recipients before FMT initiation.

a Diagram of microbial loads in PBS-treated and DHEA-treated donors and antibiotic-treated recipients.

b Relative DHEA-S levels in the fecal samples from PBS-treated and DHEA- treated groups measured by untargeted metabolomic.

c Quantification of DHEA-S levels in two groups of donors using LCMSMS. n=6 each group.
