# Supplementary material for: Androgen-induced gut dysbiosis disrupts glucolipid metabolism and endocrinal functions in polycystic ovary syndrome
Source: Microbiome. 2021 May 6;9:101. doi: 10.1186/s40168-021-01046-5 (PMC8103748; doi:10.1186/s40168-021-01046-5)
Supplement: Supplementary file 6 — Additional file 5: Figure S5. Effects of DHEA-shaped gut microbiome transplantation in recipient rats. [file 40168_2021_1046_MOESM6_ESM.pdf]

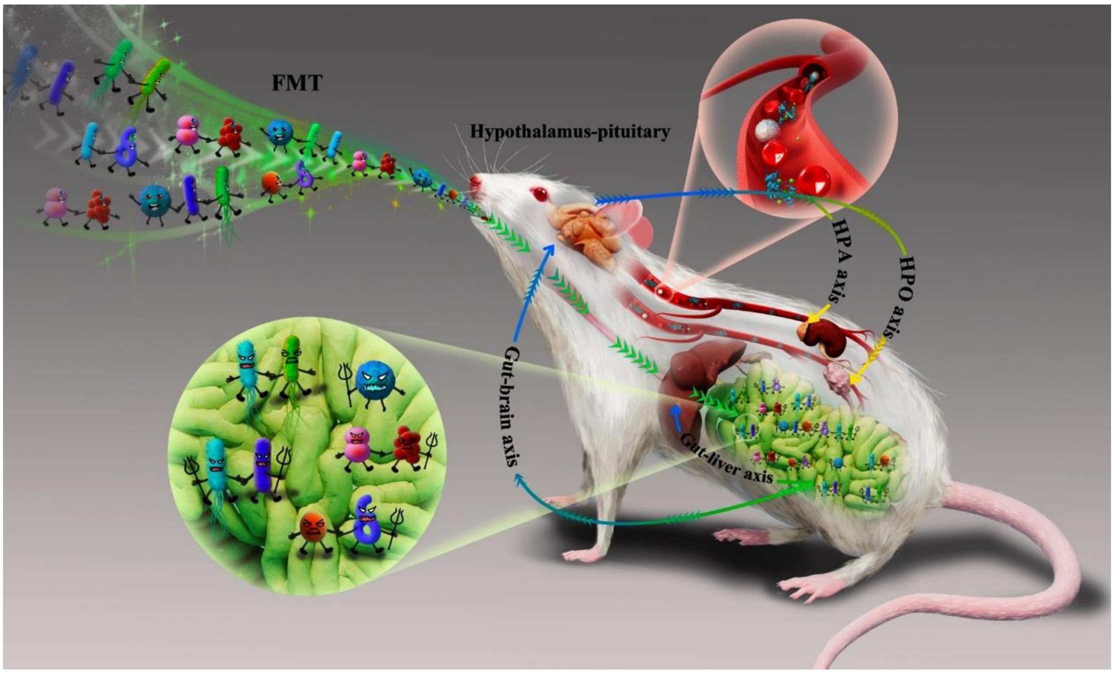

Figure S5. Effects of DHEA-shaped gut microbiome transplantation in recipient rats

Following FMT from PCOS-like rats, recipient rats presented gut microbiota dysbiosis similar to donor rats, along with aberrant faecal metabolite levels. Altered microbial community and metabolic profile in t-DHEA rats induced metabolic anomaly, endocrinal malfunction and polycystic ovarian morphology. Hepatic gene expression showed varied glucolipid metabolism following androgen-shaped gut microbiota transplantation, which may contribute to glucose intolerance, hepatic lipid accumulation and dyslipidemia. These findings implicate the potential communication through gut-liver axis. Hypothalamus also receives information conveyed through gut-brain axis. As the commander of multiple endocrinal axes, hypothalamus regulates HPO axis including gonadotropin levels and HPA axis. Perturbed HPA axis can promote androgen production by adrenal gland and interrupted functioning of HPO axis. Gonadotropin imbalance, hyperandrogenism, and metabolic disorders coordinate to induce disrupted oestrous cycles and polycystic ovary morphology.
